# Communication in Education – Improving Student Engagement With Mental Health Online Learning

**DOI:** 10.1192/bjo.2024.330

**Published:** 2024-08-01

**Authors:** Yuki Takao, Sophie Butler

**Affiliations:** 1 South London and Maudsley, London, United Kingdom; 2 King's College London, London, United Kingdom

## Abstract

**Aims:**

Our mental health placement has a longitudinal design and students spend one day per week in psychiatry throughout their academic year, attending clinical placement and academic days on alternate weeks. Academic days include a small group tutorial, Balint group and self-directed learning, during which students are expected to complete a ‘virtual patient’ (VP) e-learning module. VPs align with the topic of their small group tutorial and are intended to facilitate their learning. Despite this, only a minority of students complete them. Our aim is to investigate and iteratively design interventions to improve uptake of learning.

**Methods:**

Using an iterative process, we explored potential reasons for low student uptake by reviewing routine feedback from faculty and students; most tutors and students were unaware of the e-learning. We hypothesised that increased faculty awareness and promotion of VP could lead to an increase in student access numbers. The intervention was therefore of improved communication amongst faculty by fortnightly newsletter emails which include the topic of the academic day, explanation around what is expected of students with the website link to the VP, and other useful resources tutors may wish to use. Emails are sent by medical education administrators in the week before the academic day to all tutors. Access logs for the previous (2022–2023) and current (2023–2024) academic year were obtained so comparison could be made pre- and post-intervention.

**Results:**

Results are available for 4 modules; thus far we are yet to see significant differences in engagement. There was a technical glitch for one module and for the other modules the difference in student access has been minimal (<5%). We also have qualitative feedback from 5/28 tutors. One was confused and thought they were being asked to do additional work, one requested for information about the VP without realising it was included in the newsletter email, and three said they found it helpful.

**Conclusion:**

Fortnightly email newsletters is a simple and cost-effective way to possibly improve communication within faculty. It is likely difficult to promote student engagement with activities that their tutor is unaware of or perceives as invaluable. However there remain real challenges of using email as a communication tool for busy clinicians and is unlikely to make a difference as a stand-alone intervention. For future development we plan to include medical students and clinical supervisors as recipients in the mailing-list and spend time on a faculty development day to further explore this issue.

